# Paired Transcriptomic Analyses of Atheromatous and Control Vessels Reveal Novel Autophagy and Immunoregulatory Genes in Peripheral Artery Disease

**DOI:** 10.3390/cells13151269

**Published:** 2024-07-28

**Authors:** Praveen Machiraju, Rajesh Srinivas, Ramaraj Kannan, Robbie George, Stephane Heymans, Rupak Mukhopadhyay, Arkasubhra Ghosh

**Affiliations:** 1 GROW Research Laboratory, Narayana Nethralaya Foundation, Bangalore 560099, India; praveen.machi@narayananethralaya.com (P.M.); ramaraj@narayananethralaya.com (R.K.); 2 Department of Cardiology, Cardiovascular Research Institute Maastricht (CARIM), Maastricht University, 6229 ER Maastricht, The Netherlands; s.heymans@maastrichtuniversity.nl; 3 Department of Vascular and Endovascular Surgery, Narayana Health, Bangalore 560099, India; rajeshsrinivas5@gmail.com (R.S.); drrobbiegeorge@gmail.com (R.G.); 4 Centre for Molecular and Vascular Biology, Department of Cardiovascular Sciences, KU Leuven, Herestraat 49, bus911, 3000 Leuven, Belgium; 5 Department of Molecular Biology and Biotechnology, Tezpur University, Tezpur 784028, India

**Keywords:** atherosclerosis, peripheral artery disease, paired transcriptomics, metanalysis, autophagy, telomeric regulation

## Abstract

Peripheral artery disease (PAD), a significant health burden worldwide, affects lower extremities due to atherosclerosis in peripheral vessels. Although the mechanisms of PAD have been well studied, the molecular milieu of the plaques localized within peripheral arteries are not well understood. Thus, to identify PAD-lesion-specific gene expression profiles precluding genetic, environmental, and dietary biases, we studied the transcriptomic profile of nine plaque tissues normalized to non-plaque tissues from the same donors. A total of 296 upregulated genes, 274 downregulated genes, and 186 non-coding RNAs were identified. *STAG1, SPCC3, FOXQ1*, and *E2F3* were key downregulated genes, and *CD93* was the top upregulated gene. Autophagosome assembly, cellular response to UV, cytoskeletal organization, TCR signaling, and phosphatase activity were the key dysregulated pathways identified. Telomerase regulation and autophagy were identified as novel interacting pathways using network analysis. The plaque tissue was predominantly composed of immune cells and dedifferentiated cell populations indicated by cell-specific marker-imputed gene expression analysis. This study identifies novel genes, non-coding RNAs, associated regulatory pathways, and the cell composition of the plaque tissue in PAD patients. The autophagy and immunoregulatory genes may drive novel mechanisms, resulting in atheroma. These novel interacting networks and genes have potential for PAD-specific therapeutic applications.

## 1. Introduction

Peripheral artery disease (PAD) is a progressive atherosclerotic disease affecting large peripheral arteries in humans often localized mainly to branch sites of the artery [1]. PAD is one of the leading causes of morbidity due to atherosclerosis after coronary artery disease (CAD) and stroke [2]. The prevalence of PAD increases considerably with age, accounting for 1% of the total global deaths due to disease [1,3]. PAD strongly correlates with the occurrence of cardiovascular events, thus contributing to an enormous economic burden in both developed and developing nations [4,5]. 

Atherosclerotic lesions are complex, involving vascular endothelial and smooth muscle cells with dysregulated gene expression, leading to interactions with monocytes and other immune cell types [6,7]. The altered secretory and behavioral properties of these cells along with the accumulation of LDL in the intimal space lead to plaque formation [8,9]. Although the sequence of atherosclerotic events has been elucidated, the molecular pathophysiology of PAD has not been well elucidated. Although recent reports on transcriptional analyses have identified altered immune- and inflammation-related pathways, such studies have used blood samples or control samples from arterial beds of non-disease subjects [10,11]. The literature on transcriptomic studies of PAD is relatively limited compared with that of coronary artery disease or other types of vascular disease. Even though both CAD and PAD are manifestations of atherosclerosis in large arteries, significant differences in both presentation and molecular pathology exist between the two diseases, making it imperative to further understand the molecular phenotype of atherosclerosis in PAD patients [2,11,12]. 

In this study, we identified localized dysregulated genes that might contribute to the pathogenesis and progression of atherosclerosis in peripheral artery disease, and hence, we compared plaque material and non-atheromatous control vascular tissues from the same PAD patients. Our method of sample collection not only controlled for genetic variations but also nullified gender, risk factor exposure, diet, and location-based differences in atherogenesis. This allowed for the identification of genes and pathways that are directly involved and dysregulated in atherosclerotic plaques in patients. Further, a comprehensive transcriptomic analysis was performed to determine non-coding RNAs and cellular subtypes.

## 2. Materials and Methods

### 2.1. Sample Collection and Processing

Twenty pairs of non-diseased control samples and atherosclerotic plaque samples were collected from subjects of peripheral artery disease undergoing femoral artery bypass surgery at Narayana Institute of Vascular Sciences after obtaining informed consent and institutional guidelines (IRB ethics approval no. NHH-MEC-CL-2015-355). The samples were immediately processed for RNA isolation and tissue fixation in formalin for staining. Samples with very low RNA integrity scores (RIN) and library sizes were excluded. A total of 9 samples out of 20 passed quality check and were used for further transcriptomic analysis. The cohort characteristics are listed in Table S1. 

### 2.2. RNA Isolation and Quantitative Real-Time PCR

Total RNA was isolated from control and plaque tissues using the TRIzol method according to manufacturer’s protocol (Invitrogen, Carlsbad, CA, USA). Briefly, the tissues were suspended in 500 µL TRIzol reagent, vortexed for 15 s, and incubated at room temperature for not more than 2 min. 200 µL of chloroform was added for phase separation, followed by centrifugation at 13,200 rpm for 10 min. The upper aqueous layer was then added to a fresh tube containing 500 µL of isopropyl alcohol and incubated for RNA precipitation at −40 °C for 30 min. The precipitated RNA was centrifuged at 13,200 rpm for 15 min, washed twice with 70% alcohol, and suspended in RNAse-free water. The concentration and purity of the extracted mRNA were assessed using a spectrophotometer (Eppendorf Spectrophotometer Plus, Eppendorf, Hamburg, Germany). cDNA conversion was performed using the Bio-Rad iSCRIPT cDNA synthesis kit (Bio-rad, Hercules, CA, USA). Quantitative real-time PCR was performed using an SYBR green reagent (Kapa Biosystems Inc., Willmington, MA, USA). The quantitative real-time PCR (qRT-PCR) cycle included pre-incubation at 95 °C for 3 min and 40 amplification cycles at 95 °C for 10 s and 58 °C for 30 s using a CFX ConnectTM real-time PCR detection system (Bio-Rad, Hercules, CA, USA). A list of genes validated and corresponding to primer sequences is given in Table S8. 

### 2.3. RNA Sequencing and Data Analysis

RNA sequencing was performed using the Illumina HiSeq platform (Illumina Inc., San Diego, CA, USA). Data analysis was carried out using a standard bioinformatic pipeline for transcriptomic analysis. The analysis pipeline included trimming of the adapters and removing bases with low quality (Phred score > 30%), followed by contamination removal (rRNA, tRNA, mitochondrial sequences, etc.) using Trimmomatic (version-0.36) and Bowtie (version 2.2.4). The pre-processed reads were then aligned to the human genome (hg19) using HISAT2 (version-0.1.7). The reads aligned were used for finding differential gene expression (DGE) using Feature Counts (version-1.5.2) and DeSeq2. 

### 2.4. Differential Gene Expression Calculation

The raw read counts for control and case samples were normalized using DESeq2 with an adjusted *p*-value > 0.99 and a *p*-value < 0.05. log2 (foldchange), which were found to be normally distributed. From this distribution, the genes that were found to be 2 standard deviations away from the mean (mean ± 2SD) were considered as differentially expressed. The downstream annotation was performed using these differentially expressed genes. Gene ontology annotations were obtained using AmiGO2. 

### 2.5. Tissue Processing and Staining

The tissue sample from patients was immediately fixed in formalin. The tissues were then embedded in paraffin wax, and 4 µm sections were taken using a Leica microtome 2235 (Leica Microsystems, Wetzlar, Germany). The sections were kept on a hot plate for 1 h at 65 °C and processed for hematoxylin and eosin staining (H&E) staining. For H&E staining, the tissue was hydrated using xylene and alcohol for 5 min each. Tissues were stained with Harris’s hematoxylin for 10 min, followed by washes in acid alcohol and 2% sodium bicarbonate for 2 min each. Eosin staining was performed for 1 min. The tissues were hydrated by washing in alcohol and xylene for 2 min each and mounted using a DPX mounting medium.

### 2.6. Bio-Informatic Analysis and Visualization

Data visualization and gene network analysis were performed using Orange (version-3.36.1) [13] and KEGG pathway analysis, respectively. The miRNA identification and differential expression quantification of non-coding RNA were performed using miR Master 2.0 [14]. Cell marker 2.0 was used for cell imputations [15]. Cell enrichment scores were obtained from dividing the number of identified gene markers with the number of gene markers available in the database. Gene networks and gene enrichment network visualization were performed in Cytoscape (version 3.10.1) using ClueGO [16,17]. Transcription factor enrichment analysis was performed using ChEA3 [18]. Meta-analysis was performed by manually searching through the GEO and EMBL-EBI databases.

## 3. Results

### 3.1. Transcriptomic Profiling Reveals Novel Dysregulated Genes in PAD

**Figure 1:**
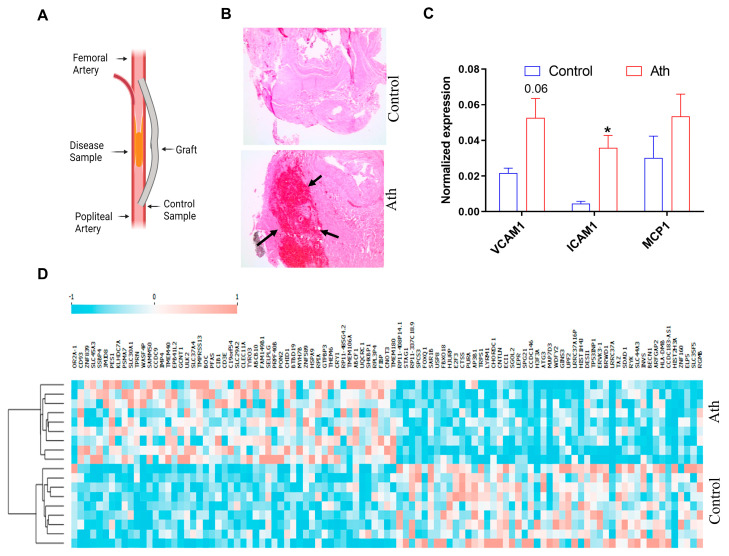
RNA sequencing from atheromatous tissues obtained from subjects undergoing femoro-popliteal bypass surgery shows altered gene expression profiles. (**A**) Schematic shows the site of sample collection. (**B**) H&E staining showing atheromatous tissue as compared with control (atheromatous plaque sample). Arrows indicate a plaque region of the sample marked by dense eosin staining. (**C**) Normalized gene expression profiles of *ICAM1, VCAM1*, and *MCP1* in atheromatous tissue (Ath) and control tissue, n = 3, * *p* < 0.05, Mann–Whitney U test. (**D**) Heatmap of top differentially regulated genes identified through RNA sequencing. Each column of the heatmap represents one sample. Genes are clustered using k-means clustering.

To validate our sample collection method (Figure 1A), we performed H&E staining to confirm that the controls used in this study were non-atherosclerotic. Dense eosin staining of a plaque in the atherosclerotic tissue sample was observed but not in controls (Figure 1B). In addition, the gene expressions of atherosclerotic markers such as intracellular adhesion molecule-1 (*ICAM*), vascular cell adhesion molecule-1 (*VCAM*), and monocyte chemoattractant protein-1 (*MCP-1*) were assessed using qRT-PCR. *ICAM1* (FC = 9.23, *p* < 0.05) showed significant upregulation, while *VCAM1* and *MCP-1* showed a 2.45-fold and 1.7-fold higher expression trend, respectively, in atherosclerotic tissues, confirming that the molecular expression patterns between the matched disease and control tissues from patients were identified correctly (Figure 1C). 

A total of 296 significantly downregulated and 274 upregulated genes were identified in the combined RNA sequencing analysis of nine paired samples. Figure 1D shows a heatmap of the top differentially regulated genes identified in this study. The top downregulated genes were *STAG1* (log2FC = −0.63, *p* < 0.05), *SPCS3* (log2FC = −0.62, *p* < 0.05), *SAR1B* (log2FC = −0.61, *p* < 0.05), and *USP8* (log2FC = −0.61, *p* < 0.05). The top downregulated transcription factors comprised *FOXQ1* (log2FC = −0.61, *p* < 0.05) and *E2F3* (log2FC = −0.61, *p* < 0.05). *E2F3* has been implicated in atherosclerosis [19], but the role of FOXQ1 in atherosclerosis is unknown.

The top upregulated genes identified included immune-related and cell surface adhesion genes like *CD93* (log2FC = 0.76, *p* < 0.05), JMJD8 (log2FC = 0.6, *p* < 0.05), and *PES1* (log2FC = 0.61, *p* < 0.05). The zinc finger protein encoded the gene *ZNF839* (log2FC = 0.72, *p* < 0.05), solute receptor family member *SLC45A3* (log2FC = 0.71, *p* < 0.05), and *SSBP4* (log2FC = 0.68, *p* < 0.05). *CD93* is a well-known transmembrane receptor and is important in promoting monocyte adhesion and further aiding in macrophage migration [20]. Table S2 lists the top dysregulated genes identified through transcriptomic analysis. Gene expression analysis using quantitative real-time PCR validated findings from RNA-seq analysis. Genes such as *AP3B1, LYRM1, USP8*, and *Rab11* were found to be downregulated in transcriptomic data as well as gene expression analysis in the plaque tissues. Meanwhile, genes such as *FGFR3, LOXL1, and ULK2* were found to be upregulated (Figure S1).

### 3.2. Gene Ontology Analysis Identifies Key Pathways in PAD

**Figure 2:**
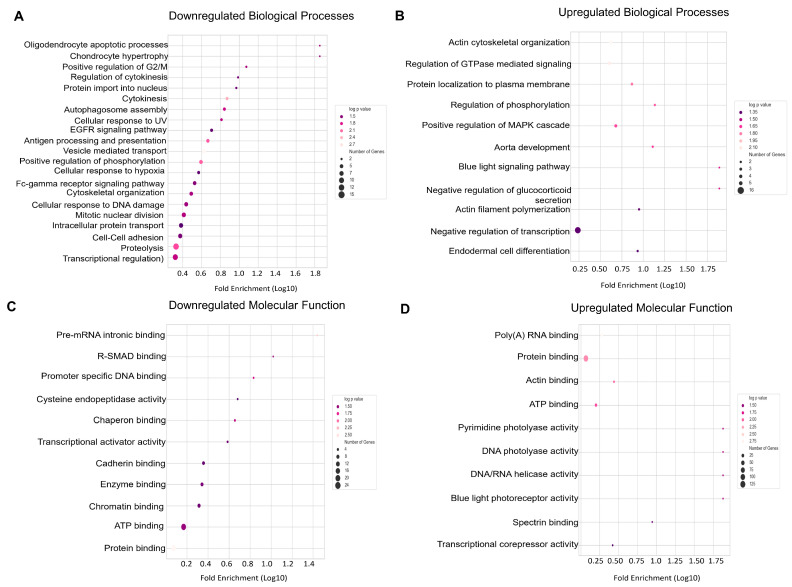
Altered biological processes associated with PAD. (**A**) Downregulated biological processes in PAD. (**B**) Upregulated biological processes. (**C**) Downregulated molecular functions. (**D**) Upregulated molecular functions associated with PAD. The color scale in bubble plots indicates a log *p*-value, the size of the bubble denotes number of genes represented, and X-axis represents the log10 value of fold enrichment.

**Figure 3:**
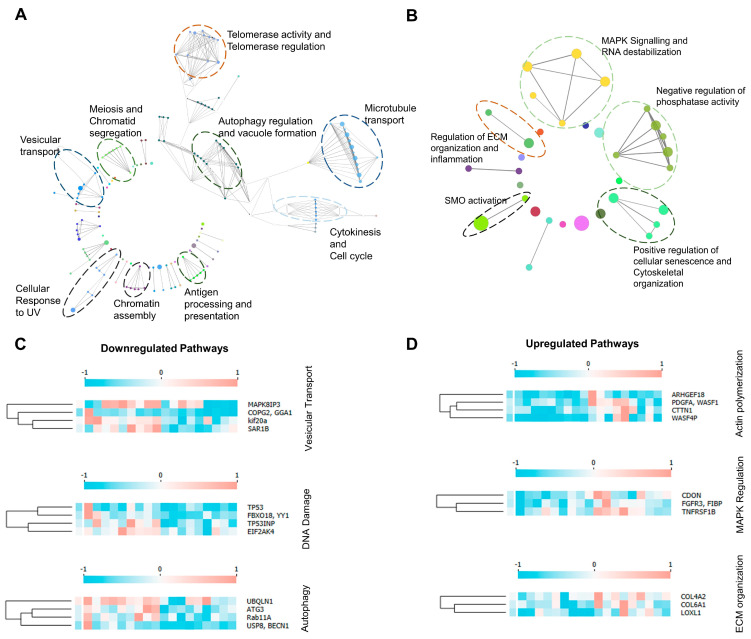
Network analysis identifies unique pathway interactions in PAD. (**A**) Downregulated pathway networks in PAD; encircled network interactions represent novel interactions found in PAD. (**B**) Upregulated pathway networks in PAD; encircled network interactions represent novel pathways identified in PAD. (**C**) Heatmaps show the relative fold change of downregulated genes specific to the identified pathways from the dataset. (**D**) Heatmaps show the relative fold change of upregulated genes specific to the identified pathways from the dataset. Columns indicate genes, and rows indicate independent samples. For heatmaps, gene expression is normalized with the average gene expression value, and genes with a similar k-mean are clustered together.

Key downregulated biological processes comprised autophagosome assembly (FE = 6.97, *p* < 0.05), vesicle mediated transport (FE = 4.23, *p* < 0.05), and cellular response to DNA damage (FE = 2.74, *p* < 0.05) (Figure 2A, Table S4). Autophagosome assembly and vesicle transport included genes such as *BECN1, ATG3, RAB11A, UBQLN1*, and *SAR1B*. Upregulated biological processes included enrichment of actin cytoskeleton organization (FE = 4.18, *p* < 0.05) and actin filament polymerization (FE = 8.9, *p* < 0.05) (Figure 2B). The pathways identified are indicative of multiple dysregulated pathways primarily related to endothelial cells and macrophages, both crucial drivers of disease progression. Upregulated pathways also included focal adhesion genes, MAPK signaling and TCR signaling (Figure S2B, Table S3). Downregulated molecular functions included pre-mRNA intronic binding (FE = 27.5, *p* < 0.05) and R-SMAD binding (FE = 10.48, *p* < 0.05) (Figure 2C), and upregulated molecular functions were DNA photolyase (FE = 76, *p* < 0.05), DNA helicase activity (FE = 76, *p* < 0.05), etc. (Figure 2D, Table S3). Overall, the gene ontology from upregulated genes indicated a pro-inflammatory milieu and a significantly higher actin polymerization. Pathway enrichment network analyses revealed novel pathway interactions such as telomerase regulation and autophagy regulation (Figure 3A). The pathways identified using upregulated genes were found to be independent of each other (Figure 3B). Figure 3C,D show differentially expressed genes in the pathways identified. 

### 3.3. Gene Networks Involved in PAD Pathogenesis Reveal Novel Interactions 

**Figure 4:**
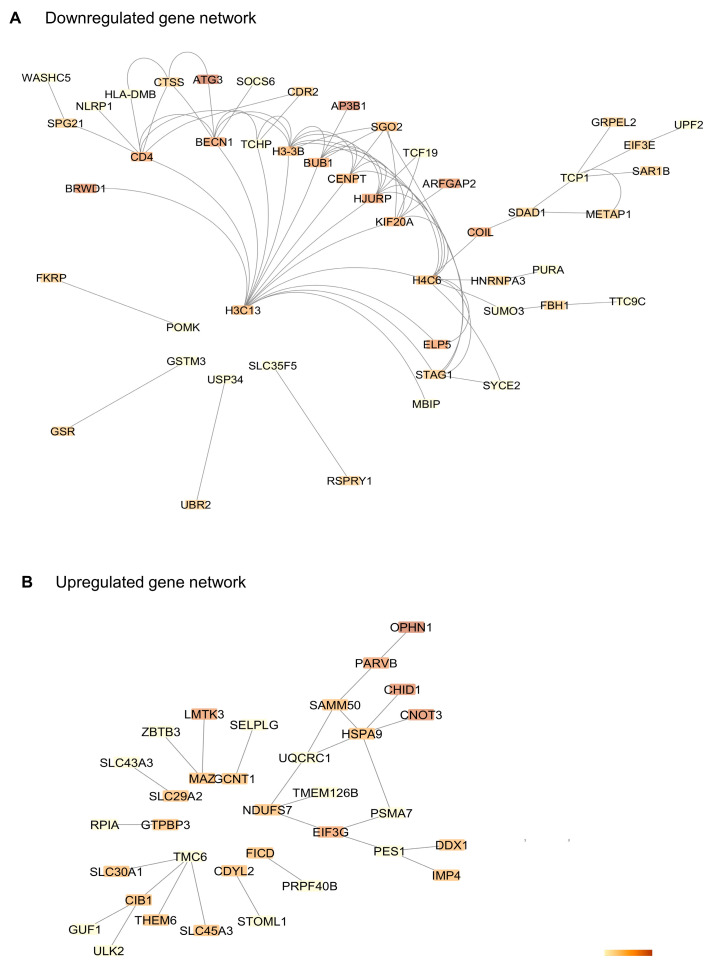
Gene network interactions in PAD. (**A**) Downregulated gene network of top 100 downregulated genes in PAD showing H3C13 having the most interactions. (**B**) Upregulated gene network of the top 100 upregulated genes in PAD. Gene network interactions were studied using STRING and visualized using Cytoscape. The color scale represents the shortest average path length.

**Figure 5:**
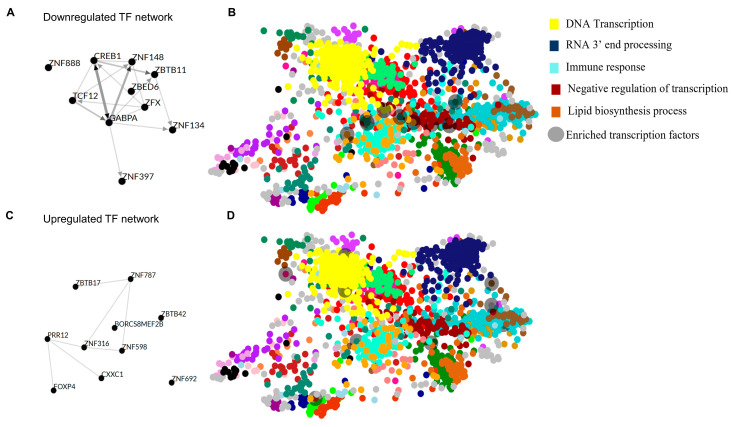
Novel transcription factor networks in PAD. (**A**) Transcription factor network analysis of the top 10-ranked transcription factors from downregulated genes identified in plaque samples compared with controls. (**B**) Downregulated transcription factors overlapped to color maps obtained from the Genotype-Tissue Expression TF network (GTEx TF) clustered according to GO enrichment. (**C**) Transcription factor network analysis of the top 10 ranked-transcription factors from upregulated genes identified in plaque samples compared with controls as analyzed using ChEA3. (**D**) Upregulated transcription factors overlapped to color maps obtained from Genotype-Tissue Expression TF network (GTEx TF) clustered according to GO enrichment.

The search tool for the retrieval of interacting genes (STRING) (https://string-db.org) was used to construct gene networks. Cytoscape software version 3.10.0 was used to visualize the networks. Upregulated gene network analysis had 86 nodes and 91 edges with average number of neighbors being 2116, and downregulated gene network analysis had 196 nodes, 452 edges, and an average number of neighbors of 4612. Gene network analysis using the STRING database of the top 100 downregulated genes predicted novel interactions among genes such as *CD4* and *Beclin1* as well as *CTSS*, which is known to be impaired in atherosclerosis [21]. *H4C6*, a histone coding gene, interacted with STAG1, KIF20A, and other histone protein coding genes such as *H3-3B* (Figure 4A). Solute carrier family genes such as *SLAC30A1, TMC6*, and *SLAC45A3* clustered together in the upregulated gene network analysis. Mitochondrial and metabolism-related genes such as *NDUFS7, TMEM126B*, and *EIF3G* were clustered together, indicating a metabolic impairment in atherosclerosis (Figure 4B). Together, the gene interaction network analysis of downregulated genes predicted novel networks relating to autophagy and T-lymphocyte function as well as interactions among histone protein coding genes and genes related to DNA stability. Novel interactions of mitochondrial genes with cytoskeletal genes such as *PARVB* were observed in the downregulated set. Downregulated TFs (transcription factors) enriched in the dataset included *CREB1, GABPA,* and *TCF12* (Figure 5A), and the upregulated TF network included *ZNF316* and *FOXP4* (Figure 5C). Most of the downregulated transcription factors in the network pertained to immune response and lipid biosynthesis (Figure 5B), while bioprocesses like negative regulation of transcription, DNA transcription, and RNA3′ processing were upregulated (Figure 5D). 

### 3.4. Non-Coding RNA Analysis Identified Novel Non-Coding RNAs in PAD 

**Figure 6:**
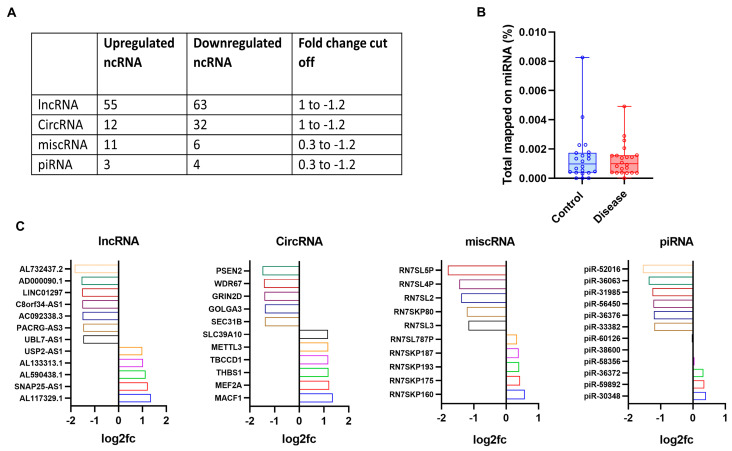
Non-coding RNAs in PAD provide insights to underlying dysregulated mechanisms. (**A**) The table shows the number of upregulated and downregulated non-coding RNAs identified along with the respective fold change cut off values used in the study. (**B**) The percentage of sequences from RNA-sequencing mapped to the miRNA database. (**C**) Bar plots showing a differential expression of non-coding RNAs as identified using miR Master v2.0 in a PAD cohort.

Four types of regulatory ncRNA, namely, long non-coding RNA (lncRNA), circular RNA (circRNA), miscellaneous RNA (miscRNA), and piwi-interacting RNA (piRNA), were mapped in the dataset using miR Master v2.0. A total of 118 dysregulated lncRNAs, 44 dysregulated circRNAs, 17 dysregulated miscRNAs, and 7 dysregulated piRNAs were identified (Figure 6A). We could not detect a significantly differentially expressed miRNA since only 0.001% of the sequences could be mapped to an existing miRNA database (Figure 6B). Most of the non-coding RNAs (ncRNA) identified in the study were novel to PAD. Since studies on ncRNA in PAD are sparse, ncRNA might be unique to the current study. CircRNA from the *MACF1* (Log2FC = 1.36) and *MEF2A* (Log2FC = 1.2) loci were upregulated, whereas those from the *PSEN2* (Log2FC = −1.47) and *WDR67* (Log2FC = −1.42) loci were downregulated. CircRNAs regulate cellular behavior by sponging up the target miRNAs, which in turn usually upregulate the mRNA targets of the corresponding miRNAs. However, since the corresponding miRNAs potentially regulated by these circRNAs could not be identified accurately, the correlation of the direct miRNA targets of these circRNAs was not performed in our data. AL117329.1(Log2FC = 1.3, *p* < 0.05) and AL732437.2 (Log2FC = −1.8, *p* < 0.05) were the top upregulated and downregulated lncRNAs, respectively (Figure 6C) (Table S5). 

### 3.5. Cell Profiling Predicts Key Cell Types Involved in PAD

**Figure 7:**
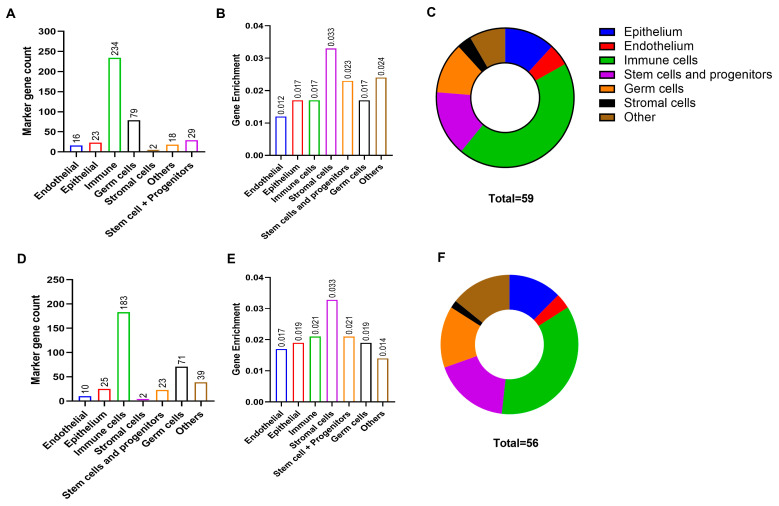
Cellular composition of PAD plaques. (**A**) Marker gene counts, (**B**) gene enrichment, and (**C**) major cell types identified and their proportions imputed using downregulated genes. (**D**) Marker gene counts, (**E**) gene enrichment, and (**F**) major cell types identified and their proportions imputed using upregulated genes. Gene enrichment was calculated as a ratio of the number genes mapped to a specific cell type and the total number of cell-specific genes.

To understand the cellular subtypes that differentially regulated genes could be attributed to in PAD, we imputed cell fractions in PAD from the list of upregulated and downregulated genes using the CellMarker 2.0 database. Figure 7A shows downregulated genes mapped to different cell types, and Figure 7D shows upregulated genes mapped to specific cell types. A total of 59 contributing cell types were identified within the downregulated gene list (Figure 7C), and 56 cell types in the upregulated gene list (Figure 7F, Table S6). The cell types were then divided into seven categories depending on their biological function. Stromal cell markers were enriched with a gene enrichment of 0.033 in both upregulated and downregulated gene lists (Figure 7B,E). Immune cell markers were the highest mapped gene markers in both upregulated and downregulated genes, 183 and 234, respectively (Table S7).

### 3.6. Meta-Analysis Identifies Unique and Common Genes in PAD 

**Figure 8:**
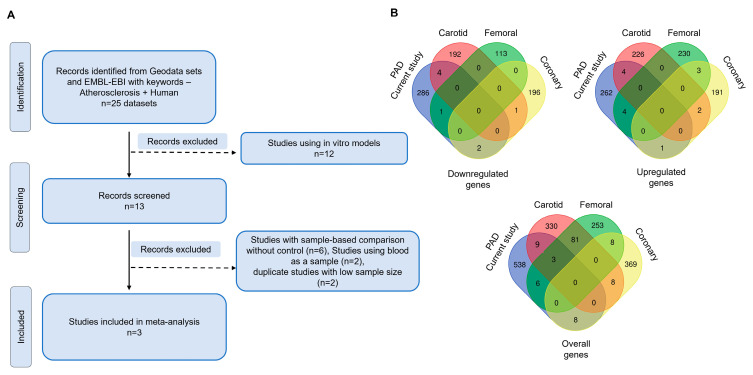
Meta-analysis across atherosclerosis studies: (**A**) Flowchart showing the methodology for the screening of available gene expression datasets using ‘atherosclerosis and human’ as keywords. A total of 4 datasets were used for meta-analysis after screening. (**B**) Venn diagram showing common and unique genes across this study (PAD_Current study), carotid artery atheroma (Carotid) (GSE43292), femoral artery disease (Femoral) (Tampere Vascular Study, 2017), and coronary artery disease (Coronary) (E-GEOD-40231). Venn diagrams were generated using bioinformatics.psb.ugent.be/webtools/Venn (accessed on 14 June 2024).

We compared our data with existing transcriptomic data from different arterial beds. Gene expression profiling data of atheroma tissues were obtained from the Gene Expression Omnibus (GEO) database and the EMBL-EBI database. A search query using ‘Atherosclerosis and Humans’ yielded 25 gene expression datasets. Expression studies from in-vitro models using blood samples and with inadequate data and no disease controls were excluded. Three datasets were included in the study after filtering for datasets with similar readouts and data availability to compare gene expression profiles from carotid atheroma (GSE43292), femoral artery disease (Tampere Vascular Study), and coronary atheroma (E-GEOD-40231) (Figure 8A). Among the downregulated genes, the current study had 4 genes in common with carotid atheroma (*CD4, TNFRSF21, CD180, MME*), 1 gene in common with femoral artery disease (*SLC22A3*), and 2 genes in common with coronary atheroma (*RGMB, UNC45A*). A total of 286 downregulated genes were found to be unique to our study. No genes were found to be common across all the studies. Four upregulated genes were found to be common between this study and carotid atheroma (HLF, SYDE2, PAK3, ADAMTSL3) as well as between this study and femoral artery disease (KRT18, IL1RN, HLA-DRB1, LMNA). One gene was found common between coronary atheroma and this study (ZC3H3). A total of 262 upregulated genes were unique to the current study. A total of 538 genes were unique to this study irrespective of the differential regulation. Common genes included SLC22A3, TNFRSF21, IL1RN in this study, carotid artery, and femoral artery disease (Figure 8B). 

## 4. Discussion

Comparative molecular studies designed to understand complex disease phenotypes typically involve healthy donors and patients where a critical limitation is that the basal expression levels of genes and the genetic makeup between controls and patients do not always match. Therefore, transcriptomic analysis was performed by comparing the gene expression profiles of the atheromatous peripheral artery with a matched non-affected patent artery sample of the same patient. The genes identified in such paired analysis from patients can be held in greater confidence for their involvement in disease progression as the study design negates the identification of those genes that might be differentially expressed elsewhere in the vasculature due to age-related, genetic, metabolic, or comorbid conditions of the patients, affecting broader changes in physiology. This study not only provides a novel approach to better identify disease-related genes but also is one of the first reports from a south Indian patient cohort where molecular expression phenotypes were not investigated previously.

Differentially expressed genes such as *CD93, SAR1B*, and *USP8* detected using such paired comparisons are novel discoveries in PAD. SAR1B functions as a transmembrane receptor for lipids and is important for maintaining lipid homeostasis. Mutations in *SAR1B* may cause chylomicron retention disease [22,23]. FOXQ1 is a transcription factor involved in invasion and metastasis through the EGF receptor pathway [24]. Although many downregulated genes are relevant to atherosclerosis, their role in the molecular mechanisms of the disease needs further studies. *CD93*, which was upregulated in PAD atheroma, is a cell surface lectin receptor expressed on macrophage and endothelial membranes. CD93 is crucial for intracellular adhesion and the clearance of apoptotic cells [25]. In endothelial cells, CD93 promotes cell–cell adhesion through beta integrin activation and fibrillogenesis, leading to angiogenesis [26]. *SLC45A3*, which was also elevated in atheroma, plays an important role in lipid metabolism [27]. The downregulation of *STAG1* in PAD may be associated with genomic stability or predispose tissues to DNA damage since mutations in *STAG1* have been reported to predispose children to hematological malignancies [28]. *USP8*, altered in our data, is important for endosomal trafficking in atherosclerosis and is induced in the presence of growth arrest during cell–cell contact and is also important in RAS signaling and Wnt pathway regulation [29]. Existing gene expression studies in PAD have identified genes like *MMP9, MMP12, SPP1*, and *APOD;* however, these identifications compare gene expressions in plaques from different arterial beds and not from the same subject [11]. The pathways identified in previous studies are also predominantly of immune- and inflammation-related pathways [30]. Other studies either targeted specific cells such as macrophages or the identifications were not from the plaque material of PAD subjects [31,32,33]. Since the dysregulated genes specific to the plaque milieu of the PAD patients are controlled to their own patent vessel tissues, many of the genes that have been identified in other studies did not show significance in our study. Such genes may be altered in the non-lesion areas of the vessels or be associated with more global changes occurring within the PAD patient. Therefore, this analysis furthers our understanding of advanced PAD and identifies these genes as specific targets for future therapies directed towards the atheromatous lesions in advanced disease.

Pathway enrichment analysis primarily identified vacuole assembly, autophagy, and EGFR signaling as key downregulated pathways apart from cellular response to UV and protein localization. TCR signaling, IL-17 signaling, ECM regulation, and phosphatase activity are upregulated in pathway network analysis. In agreement with previous findings, proteinase and peptidase expression are upregulated in femoral artery plaques [34], phosphatase activity and related networks are upregulated, and the regulation of MAPK activity by phosphatases increases foam cell formation and VSMC migration during atherosclerosis [35]. The interaction between telomerase activity and autophagy-related processes was a novel finding from our study. Rescuing autophagy has been shown to ameliorate atherosclerosis in mice [36]. Although reductions in telomerase reverse transcriptase (TERT) expression levels and telomerase activity have been implicated in atherosclerosis, definitive mechanisms are yet to be elucidated [37]. Our findings suggests that telomerase regulation and autophagy processes together might confer an antiatherogenic effect ion PAD. Moreover, pathway interaction networks also indicate a possible regulatory mechanism among genes involved in these processes.

Non-coding regulatory RNA (ncRNA), such as long non-coding RNA (lncRNA), circular RNA (circRNA), miscellaneous RNA (miscRNA), piwi-interacting RNA (piRNA), and microRNA (miRNA), play crucial roles in the expression and regulation of genes and proteins through ncRNA-DNA, ncRNA-mRNA, ncRNA-protein, and ncRNA-ncRNA interactions [38,39]. Studies to identify miRNA have focused on circulating miRNAs using whole blood or PBMCs [40,41] or studying specific PAD-related events in mouse models [42], but data on human subjects have not yet been reported. LINC01297, UBL-7 AS1, and SNAP25 AS1 are novel lncRNAs identified in this study, which are known to regulate infiltration of immune cells, cell proliferation, and vesicular transport [43,44]. Together, non-coding RNA identification indicated autophagosome disassembly, upregulation of microtubule assembly, and deregulation in immune modulation. Although we could identify miRNAs from our data, no statistically significant differentially expressed miRNAs were observed, which may be due to the study design.

The classification of the cell types showed that although immune cell types were largely represented, it was the stromal cell gene signatures that were enriched in femoral artery plaques. These findings support the notion that femoral artery plaques may be more stable owing to a denser stromal matrix. However, cell subtype prediction from RNA sequencing may be limited by the available databases and cell-specific marker information on atherosclerosis. Additional validation studies to identify cell types using immunophenotyping or immunohistological techniques need to be performed in the future. 

Upon comparing our dataset with previous publications, we found that the PAD plaques had 538 unique dysregulated genes with very limited common genes across studies. The common genes identified in previous studies included immune-related genes such as *CD4, CD180*, and *TNFRSF21*. Interestingly, *SLC22A3* was commonly dysregulated in carotid artery atheroma and femoral artery disease and in the current study. *SLC22A3* has been previously implicated in hypercholesteremia and cardiovascular disorders [45]. Our recent findings suggest that *SLC22A3* might play significant roles in the broader pathophysiology of vascular diseases. Thus, the meta-analysis of the data suggests that the genes involved in PAD are different from the atheroma of other vascular beds, therefore adding important information to this field. While this study aimed to discover PAD-associated gene alterations restricting patient specific genetic, nutritional, and demographic bias, the small sample size, limited ethnic diversity, and single-center sample acquisition are study limitations. However, the discovery of many novel alterations in genes and pathways warrants further molecular investigations in PAD. Future multi-centric studies with larger sample sizes, single-cell RNA-seq assays, correlations with plasma protein profiles, and disease modeling may help validate the novel targets for potential therapies.

In conclusion, by using paired disease–control analysis, our study uncovers key genes and interacting networks that are signatures of plaques in peripheral arteries of PAD patients. Further functional studies of the genes identified may lead to the development of targeted therapies for peripheral artery disease.

## Data Availability

The original contributions presented in this study are included in the article and Supplementary Material. Further inquiries can be directed to the corresponding authors.

## Supplementary Materials

The following supporting information can be downloaded at https://www.mdpi.com/article/10.3390/cells13151269/s1, Table S1: Study cohort characteristics; Table S2: Dysregulated genes in PAD; Table S3: Gene ontology of upregulated genes in PAD; Table S4: Gene ontology of downregulated genes in PAD; Table S5: Differentially regulated non-coding RNAs identified in PAD; Tables S6 and S7: Cell imputations and marker gene counts; Table S8: Primer sequences of genes used for data validation; Figure S1: Gene expression analysis for validation; Figure S2: Gene ontology analysis of dysregulated genes.

## References

[1] Aday A.W., Matsushita K. (2021). Epidemiology of Peripheral Artery Disease and Polyvascular Disease. Circ. Res..

[2] Narula N., Olin J.W., Narula N. (2020). Pathologic Disparities Between Peripheral Artery Disease and Coronary Artery Disease. Arterioscler. Thromb. Vasc. Biol..

[3] Song P., Rudan D., Zhu Y., Fowkes F.J.I., Rahimi K., Fowkes F.G.R., Rudan I. (2019). Global, regional, and national prevalence and risk factors for peripheral artery disease in 2015: An updated systematic review and analysis. Lancet Glob. Health.

[4] Fowkes F.G., Rudan D., Rudan I., Aboyans V., Denenberg J.O., McDermott M.M., Norman P.E., Sampson U.K., Williams L.J., Mensah G.A. (2013). Comparison of global estimates of prevalence and risk factors for peripheral artery disease in 2000 and 2010: A systematic review and analysis. Lancet.

[5] Verma S., Dhingra N.K., Bonaca M.P., Butler J., Anker S.D., Ferreira J.P., Filippatos G., Januzzi J.L., Lam C.S.P., Sattar N. (2023). Presence of Peripheral Artery Disease Is Associated With Increased Risk of Heart Failure Events: Insights from EMPEROR-Pooled. Arterioscler. Thromb. Vasc. Biol..

[6] Bjorkegren J.L.M., Lusis A.J. (2022). Atherosclerosis: Recent developments. Cell.

[7] Kim K.W., Ivanov S., Williams J.W. (2020). Monocyte Recruitment, Specification, and Function in Atherosclerosis. Cells.

[8] Tabas I., Garcia-Cardena G., Owens G.K. (2015). Recent insights into the cellular biology of atherosclerosis. J. Cell Biol..

[9] Klouche M., May A.E., Hemmes M., Messner M., Kanse S.M., Preissner K.T., Bhakdi S. (1999). Enzymatically modified, nonoxidized LDL induces selective adhesion and transmigration of monocytes and T-lymphocytes through human endothelial cell monolayers. Arterioscler. Thromb. Vasc. Biol..

[10] Newman J.D., Cornwell M.G., Zhou H., Rockman C., Heguy A., Suarez Y., Cheng H.S., Feinberg M.W., Hochman J.S., Ruggles K.V. (2021). Gene Expression Signature in Patients With Symptomatic Peripheral Artery Disease. Arterioscler. Thromb. Vasc. Biol..

[11] Sulkava M., Raitoharju E., Levula M., Seppala I., Lyytikainen L.P., Mennander A., Jarvinen O., Zeitlin R., Salenius J.P., Illig T. (2017). Differentially expressed genes and canonical pathway expression in human atherosclerotic plaques—Tampere Vascular Study. Sci. Rep..

[12] Poredos P., Cevc M., Blinc A. (2021). Characteristics of atherosclerosis in femoropopliteal artery and its clinical relevance. Atherosclerosis.

[13] Demšar J., Curk T., Erjavec A., Gorup Č., Hočevar T., Milutinovič M., Možina M., Polajnar M., Toplak M., Starič A. (2013). Orange: Data mining toolbox in python. J. Mach. Learn. Res..

[14] Fehlmann T., Kern F., Laham O., Backes C., Solomon J., Hirsch P., Volz C., Muller R., Keller A. (2021). miRMaster 2.0: Multi-species non-coding RNA sequencing analyses at scale. Nucleic. Acids. Res..

[15] Hu C., Li T., Xu Y., Zhang X., Li F., Bai J., Chen J., Jiang W., Yang K., Ou Q. (2023). CellMarker 2.0: An updated database of manually curated cell markers in human/mouse and web tools based on scRNA-seq data. Nucleic. Acids. Res..

[16] Bindea G., Mlecnik B., Hackl H., Charoentong P., Tosolini M., Kirilovsky A., Fridman W.H., Pages F., Trajanoski Z., Galon J. (2009). ClueGO: A Cytoscape plug-in to decipher functionally grouped gene ontology and pathway annotation networks. Bioinformatics.

[17] Shannon P., Markiel A., Ozier O., Baliga N.S., Wang J.T., Ramage D., Amin N., Schwikowski B., Ideker T. (2003). Cytoscape: A software environment for integrated models of biomolecular interaction networks. Genome Res..

[18] Keenan A.B., Torre D., Lachmann A., Leong A.K., Wojciechowicz M.L., Utti V., Jagodnik K.M., Kropiwnicki E., Wang Z., Ma’ayan A. (2019). ChEA3: Transcription factor enrichment analysis by orthogonal omics integration. Nucleic. Acids. Res..

[19] Prestel M., Prell-Schicker C., Webb T., Malik R., Lindner B., Ziesch N., Rex-Haffner M., Roh S., Viturawong T., Lehm M. (2019). The Atherosclerosis Risk Variant rs2107595 Mediates Allele-Specific Transcriptional Regulation of HDAC9 via E2F3 and Rb1. Stroke.

[20] Tossetta G., Piani F., Borghi C., Marzioni D. (2023). Role of CD93 in Health and Disease. Cells.

[21] Wang H., Jiang H., Cheng X.W. (2022). Cathepsin S are involved in human carotid atherosclerotic disease progression, mainly by mediating phagosomes: Bioinformatics and in vivo and vitro experiments. PeerJ.

[22] Sane A., Ahmarani L., Delvin E., Auclair N., Spahis S., Levy E. (2019). SAR1B GTPase is necessary to protect intestinal cells from disorders of lipid homeostasis, oxidative stress, and inflammation. J. Lipid Res..

[23] Simone M.L., Rabacchi C., Kuloglu Z., Kansu A., Ensari A., Demir A.M., Hizal G., Di Leo E., Bertolini S., Calandra S. (2019). Novel mutations of SAR1B gene in four children with chylomicron retention disease. J. Clin. Lipidol..

[24] Zhang J.J., Cao C.X., Wan L.L., Zhang W., Liu Z.J., Wang J.L., Guo Q., Tang H. (2022). Forkhead Box q1 promotes invasion and metastasis in colorectal cancer by activating the epidermal growth factor receptor pathway. World J. Gastroenterol..

[25] Norsworthy P.J., Fossati-Jimack L., Cortes-Hernandez J., Taylor P.R., Bygrave A.E., Thompson R.D., Nourshargh S., Walport M.J., Botto M. (2004). Murine CD93 (C1qRp) contributes to the removal of apoptotic cells in vivo but is not required for C1q-mediated enhancement of phagocytosis. J. Immunol..

[26] Barbera S., Raucci L., Lugano R., Tosi G.M., Dimberg A., Santucci A., Galvagni F., Orlandini M. (2021). CD93 Signaling via Rho Proteins Drives Cytoskeletal Remodeling in Spreading Endothelial Cells. Int. J. Mol. Sci..

[27] Shin D., Howng S.Y., Ptacek L.J., Fu Y.H. (2012). miR-32 and its target SLC45A3 regulate the lipid metabolism of oligodendrocytes and myelin. Neuroscience.

[28] Saitta C., Rebellato S., Bettini L.R., Giudici G., Panini N., Erba E., Massa V., Auer F., Friedrich U., Hauer J. (2022). Potential role of STAG1 mutations in genetic predisposition to childhood hematological malignancies. Blood Cancer J..

[29] Tang J., Long G., Xiao L., Zhou L. (2023). USP8 positively regulates hepatocellular carcinoma tumorigenesis and confers ferroptosis resistance through beta-catenin stabilization. Cell Death Dis..

[30] Fu S., Zhao H., Shi J., Abzhanov A., Crawford K., Ohno-Machado L., Zhou J., Du Y., Kuo W.P., Zhang J. (2008). Peripheral arterial occlusive disease: Global gene expression analyses suggest a major role for immune and inflammatory responses. BMC Genom..

[31] Huang H.M., Jiang X., Hao M.L., Shan M.J., Qiu Y., Hu G.F., Wang Q., Yu Z.Q., Meng L.B., Zou Y.Y. (2019). Identification of biomarkers in macrophages of atherosclerosis by microarray analysis. Lipids Health Dis..

[32] Ijas P., Nuotio K., Saksi J., Soinne L., Saimanen E., Karjalainen-Lindsberg M.L., Salonen O., Sarna S., Tuimala J., Kovanen P.T. (2007). Microarray analysis reveals overexpression of CD163 and HO-1 in symptomatic carotid plaques. Arterioscler. Thromb. Vasc. Biol..

[33] Gwon S.Y., Lee H.M., Rhee K.J., Sung H.J. (2019). Microarray and proteome array in an atherosclerosis mouse model for identification of biomarkers in whole blood. Int. J. Med. Sci..

[34] Oksala N., Levula M., Airla N., Pelto-Huikko M., Ortiz R.M., Jarvinen O., Salenius J.P., Ozsait B., Komurcu-Bayrak E., Erginel-Unaltuna N. (2009). ADAM-9, ADAM-15, and ADAM-17 are upregulated in macrophages in advanced human atherosclerotic plaques in aorta and carotid and femoral arteries—Tampere vascular study. Ann. Med..

[35] Reustle A., Torzewski M. (2018). Role of p38 MAPK in Atherosclerosis and Aortic Valve Sclerosis. Int. J. Mol. Sci..

[36] Lu H., Fan Y., Qiao C., Liang W., Hu W., Zhu T., Zhang J., Chen Y.E. (2017). TFEB inhibits endothelial cell inflammation and reduces atherosclerosis. Sci. Signal.

[37] Herrmann W., Herrmann M. (2020). The Importance of Telomere Shortening for Atherosclerosis and Mortality. J. Cardiovasc. Dev. Dis..

[38] Esteller M. (2011). Non-coding RNAs in human disease. Nat. Rev. Genet..

[39] Zhang P., Wu W., Chen Q., Chen M. (2019). Non-Coding RNAs and their Integrated Networks. J. Integr. Bioinform..

[40] Bogucka-Kocka A., Zalewski D.P., Ruszel K.P., Stepniewski A., Galkowski D., Bogucki J., Komsta L., Kolodziej P., Zubilewicz T., Feldo M. (2019). Dysregulation of MicroRNA Regulatory Network in Lower Extremities Arterial Disease. Front. Genet..

[41] Ring A., Ismaeel A., Wechsler M., Fletcher E., Papoutsi E., Miserlis D., Koutakis P. (2022). MicroRNAs in peripheral artery disease: Potential biomarkers and pathophysiological mechanisms. Ther. Adv. Cardiovasc. Dis..

[42] Lee C.Y., Lin S.J., Wu T.C. (2022). miR-548j-5p regulates angiogenesis in peripheral artery disease. Sci. Rep..

[43] Cao M., Ma R., Li H., Cui J., Zhang C., Zhao J. (2022). Therapy-resistant and -sensitive lncRNAs, SNHG1 and UBL7-AS1 promote glioblastoma cell proliferation. Oxid. Med. Cell Longev..

[44] Eptaminitaki G.C., Wolff N., Stellas D., Sifakis K., Baritaki S. (2021). Long Non-Coding RNAs (lncRNAs) in Response and Resistance to Cancer Immunosurveillance and Immunotherapy. Cells.

[45] Paquette M., Bernard S., Baass A. (2019). SLC22A3 is associated with lipoprotein (a) concentration and cardiovascular disease in familial hypercholesterolemia. Clin. Biochem..

