# Supplementary material for: Paired Transcriptomic Analyses of Atheromatous and Control Vessels Reveal Novel Autophagy and Immunoregulatory Genes in Peripheral Artery Disease
Source: Cells. 2024 Jul 28;13(15):1269. doi: 10.3390/cells13151269 (PMC11312159; doi:10.3390/cells13151269)
Supplement: Supplementary file 1 [file cells-13-01269-s001.zip › Supplementary_revised/Supplementary table 1.pdf]

Patient ID	Age	Sex	Diagnosis	Procedure	Rutherford classification	Comorbidities
P1	41	M	Right lower limb critical ischemia	Right femoral distal bypass	V	None
P2	42	M	Left lower limb critical limb ischemia	Left SFA to TP trunk bypass	V	DM
P3	62	M	Right lower limb critical ischemia	Right femoral distal bypass	NA	NA
P4	72	M	Right lower limb critical limb ischemia	Right CFA-popliteal bypass	V	DM
P5	57	M	Left lower limb critical limb ischemia	Left SFA to PTA bypass	V	DM, SM
P6	59	M	Right lower limb critical limb ischemia	Right femoral-TP bypass + CFA Endarectomy	NA	HTN, DM, CAD
P7	65	M	Right lower limb critical limb ischemia	Right femoral-TP bypass	VI	HTN, SM
P8	46	M	Left lower limb critical limb ischemia	Left CFA popliteal bypass	III	HTN, DM, CAD, SM
P9	57	F	Left lower limb critical limb ischemia	Left ileofemoral bypass	III	HTN, DM

Table S1: Cohort characteristics: Age, Sex, Diagnosis, procedure performed along with Rutherford classification and comorbidity status of the participating subjects in the study. The table lists only those subjects from which transcriptomic studies have been performed. SFA – Superficial femoral artery, PTA- Posterior tibial artery, CFA – Common femoral artery, HTN -Hypertension, DM – Diabetes Mellitus, SM – Smoker, CAD – coronary artery disease, TP – Tibioperoneal, NA – Not available.
