# Supplementary material for: Paired Transcriptomic Analyses of Atheromatous and Control Vessels Reveal Novel Autophagy and Immunoregulatory Genes in Peripheral Artery Disease
Source: Cells. 2024 Jul 28;13(15):1269. doi: 10.3390/cells13151269 (PMC11312159; doi:10.3390/cells13151269)
Supplement: Supplementary file 1 [file cells-13-01269-s001.zip › Supplementary_revised/Supplementary table 2.pdf]

Upregulated Genes			Downregulated Genes		
**Gene name**	**log2FC**	**pvalue**	**Gene name**	**log2FC**	**pvalue**
CD93	0.76	4.3E-04	*STAG1*	-0.64	2.7E-03
ZNF839	0.73	8.7E-04	*SPCS3*	-0.62	3.0E-03
SLC45A3	0.72	1.0E-03	*FOXQ1*	-0.62	4.4E-03
SSBP4	0.68	1.8E-03	*SAR1B*	-0.61	1.0E-03
JMJD8	0.68	1.8E-03	*USP8*	-0.61	1.9E-03
PES1	0.67	1.9E-04	*FBXO18*	-0.61	2.0E-03
KLHDC7A	0.67	1.3E-03	*HJURP*	-0.61	2.9E-03
PSMA7	0.66	2.6E-03	*E2F3*	-0.60	1.1E-03
SLC30A1	0.63	3.7E-03	*CTSS*	-0.59	3.8E-03
TPRN	0.63	3.3E-03	*PURA*	-0.59	4.6E-03
WASF4P	0.63	1.4E-03	*AP3B1*	-0.59	2.7E-03
SAMM50	0.63	2.6E-03	*TRPS1*	-0.59	5.7E-03
CCDC9	0.63	4.2E-03	*LYRM1*	-0.58	3.7E-03
IMP4	0.61	4.6E-03	*CHORDC1*	-0.58	7.0E-03
TMEM40	0.61	4.9E-03	*CNTLN*	-0.58	8.1E-03
EPB41L2	0.61	9.7E-04	*ECI1*	-0.57	8.8E-03
GCNT1	0.59	6.9E-03	*SGOL2*	-0.57	9.8E-03
ULK2	0.59	5.5E-03	*LEPR*	-0.56	9.1E-03
SLC37A4	0.59	7.5E-03	*SPG21*	-0.56	1.1E-02
TMPRSS13	0.58	1.6E-03	*CCDC146*	-0.56	7.6E-03

Table S2: Top 20 differentially regulated genes as identified from transcriptomic analysis of atheroma samples from 9 paired samples.
