# Supplementary material for: Paired Transcriptomic Analyses of Atheromatous and Control Vessels Reveal Novel Autophagy and Immunoregulatory Genes in Peripheral Artery Disease
Source: Cells. 2024 Jul 28;13(15):1269. doi: 10.3390/cells13151269 (PMC11312159; doi:10.3390/cells13151269)
Supplement: Supplementary file 1 [file cells-13-01269-s001.zip › Supplementary_revised/Supplementary table 3.pdf]

Upregulated Biological processes	Fold Enrichment	Number of Genes	pvalue
GO:0030036~actin cytoskeleton organization	4.2	7	6.6E-03
GO:0051056~regulation of small GTPase mediated signal transduction	4.1	7	7.6E-03
GO:0090002~establishment of protein localization to plasma membrane	7.4	4	1.6E-02
GO:0042325~regulation of phosphorylation	13.7	3	2.0E-02
GO:0043410~positive regulation of MAPK cascade	4.8	5	2.0E-02
GO:0035904~aorta development	13.0	3	2.2E-02
GO:0009785~blue light signalling pathway	77.7	2	2.5E-02
GO:2000850~negative regulation of glucocorticoid secretion	77.7	2	2.5E-02
GO:0030041~actin filament polymerization	9.0	3	4.3E-02
GO:0000122~negative regulation of transcription from RNA polymerase II promoter	1.7	16	4.4E-02
GO:0035987~endodermal cell differentiation	8.6	3	4.6E-02

Upregulated Molecular functions	Fold Enrichment	Number of Genes	pvalue
GO:0044822~poly(A) RNA binding	1.9	28	1.7E-03
GO:0005515~protein binding	1.2	134	7.0E-03
GO:0003779~actin binding	2.7	10	1.1E-02
GO:0005524~ATP binding	1.6	31	1.2E-02
GO:0003904~deoxyribodipyrimidine photo-lyase activity	76.4	2	2.6E-02
GO:0003914~DNA (6-4) photolyase activity	76.4	2	2.6E-02
GO:0033677~DNA/RNA helicase activity	76.4	2	2.6E-02
GO:0009882~blue light photoreceptor activity	76.4	2	2.6E-02
GO:0030507~spectrin binding	8.8	3	4.5E-02
GO:0003714~transcription corepressor activity	2.6	7	5.0E-02

Upregulated Cellular components	Fold Enrichment	Number of Genes	pvalue
GO:0030027~lamellipodium	4.5	9	9.3E-04
GO:0005737~cytoplasm	1.3	88	1.2E-03
GO:0031012~extracellular matrix	3.2	12	1.3E-03
GO:0005925~focal adhesion	2.8	14	1.3E-03
GO:0005856~cytoskeleton	2.8	13	2.6E-03
GO:0005730~nucleolus	2.0	21	5.6E-03
GO:0005654~nucleoplasm	1.4	50	7.4E-03
GO:0071944~cell periphery	8.2	4	1.3E-02
GO:0005634~nucleus	1.2	84	1.7E-02
GO:0070062~extracellular exosome	1.4	48	2.1E-02
GO:0016020~membrane	1.4	39	2.4E-02
GO:0030425~dendrite	2.4	10	2.6E-02
GO:0001726~ruffle	4.4	10	2.6E-02

Upregulated Biological pathways	Fold Enrichment	Number of Genes	pvalue
hsa05131: Shigellosis	4.5	4	5.7E-02
hsa04510: Focal adhesion	2.4	7	6.4E-02
R-HSA-5687128: MAPK Signalling	5.0	6	6.5E-03
R-HSA-3000171: Non-integrin membrane-ECM interactions	7.4	4	1.6E-02
R-HSA-194840: Rho GTPase Cycle	3.6	6	2.6E-02
R-HSA-202424: TCR Signalling	3.7	5	4.7E-02

Table S3: Upregulated biological processes, molecular functions, cellular components and biological pathways identified using transcriptomic analysis.
