# Supplementary material for: Paired Transcriptomic Analyses of Atheromatous and Control Vessels Reveal Novel Autophagy and Immunoregulatory Genes in Peripheral Artery Disease
Source: Cells. 2024 Jul 28;13(15):1269. doi: 10.3390/cells13151269 (PMC11312159; doi:10.3390/cells13151269)
Supplement: Supplementary file 1 [file cells-13-01269-s001.zip › Supplementary_revised/Supplementary table 4.pdf]

Downregulated Biological processes	Fold enrichment	Number of Genes	pvalue
GO:0097252~oligodendrocyte apoptotic process	71.46	2	0.028
GO:0003415~chondrocyte hypertrophy	71.46	2	0.028
GO:0010971~positive regulation of G2/M transition of mitotic cell cycle	11.91	3	0.026
GO:0032465~regulation of cytokinesis	9.74	3	0.037
GO:0006607~NLS-bearing protein import into nucleus	9.32	3	0.040
GO:0000910~cytokinesis	7.44	5	0.004
GO:0000045~autophagosome assembly	6.97	5	0.019
GO:0034644~cellular response to UV	6.50	4	0.023
GO:0007173~epidermal growth factor receptor signalling pathway	5.10	4	0.043
GO:0019886~antigen processing and presentation of exogenous peptide antigen via MHC class II	4.66	6	0.009
GO:0016192~vesicle-mediated transport	4.23	8	0.001
GO:0001934~positive regulation of protein phosphorylation	3.94	7	0.009
GO:0071456~cellular response to hypoxia	3.72	5	0.045
GO:0038096~Fc-gamma receptor signalling pathway involved in phagocytosis	3.38	6	0.033
GO:0007010~cytoskeleton organization	3.11	7	0.025
GO:0006974~cellular response to DNA damage stimulus	2.75	8	0.027
GO:0007067~mitotic nuclear division	2.59	9	0.023
GO:0006886~intracellular protein transport	2.42	8	0.048
GO:0098609~cell-cell adhesion	2.37	9	0.036
GO:0006508~proteolysis	2.14	15	0.011
GO:0006357~regulation of transcription from RNA polymerase II promoter	2.11	13	0.021

Downregulated Molecular functions	Fold enrichment	Number of Genes	pvalue
GO:0097157~pre-mRNA intronic binding	27.52	3	0.005
GO:0070412~R-SMAD binding	10.49	3	0.032
GO:0001046~core promoter sequence-specific DNA binding	6.83	4	0.020
GO:0004197~cysteine-type endopeptidase activity	4.81	4	0.050
GO:0051087~chaperone binding	4.53	5	0.024
GO:0001228~transcriptional activator activity, RNA polymerase II transcription regulatory region sequence-specific binding	3.86	5	0.040
GO:0098641~cadherin binding involved in cell-cell adhesion	2.28	9	0.045
GO:0019899~enzyme binding	2.20	10	0.038
GO:0003682~chromatin binding	2.06	11	0.041
GO:0005524~ATP binding	1.47	30	0.032
GO:0005515~protein binding	1.19	142	0.002

Downregulated Cellular components	Fold enrichment	Number of Genes	pvalue
GO:0005654~nucleoplasm	1.57	59	0.0003
GO:0000139~Golgi membrane	2.51	20	0.0004
GO:0016020~membrane	1.58	47	0.0015
GO:0005737~cytoplasm	1.32	93	0.0015
GO:0005829~cytosol	1.39	62	0.0051
GO:0005730~nucleolus	1.90	22	0.0060
GO:0005789~endoplasmic reticulum membrane	1.89	22	0.0064
GO:0005794~Golgi apparatus	1.89	22	0.0065

GO:0005634~nucleus	1.23	90	0.0147
GO:0043234~protein complex	2.16	12	0.0240
GO:0005783~endoplasmic reticulum	1.70	19	0.0307
GO:0000785~chromatin	4.16	5	0.0320
GO:0030529~intracellular ribonucleoprotein complex	3.27	6	0.0368
GO:0016363~nuclear matrix	3.82	5	0.0419

**Downregulated Biological pathways**	**Fold enrichment**	**Number of Genes**	**pvalue**
hsa05416: Viral myocarditis	5.61	5	0.011
hsa04612: Antigen processing and presentation	4.21	5	0.030
hsa05222: Small cell lung cancer	3.76	5	0.042
R-HSA-2132295: MHC classII presentation	3.47	7	0.015
R-HSA-2029482: Phagocytic cup formation	3.08	6	0.045
hsa04144: Endocytosis	2.48	10	0.018

Table S4: Downregulated biological processes, molecular functions, cellular components and biological pathways identified using transcriptomic analysis.
