# Supplementary material for: Paired Transcriptomic Analyses of Atheromatous and Control Vessels Reveal Novel Autophagy and Immunoregulatory Genes in Peripheral Artery Disease
Source: Cells. 2024 Jul 28;13(15):1269. doi: 10.3390/cells13151269 (PMC11312159; doi:10.3390/cells13151269)
Supplement: Supplementary file 1 [file cells-13-01269-s001.zip › Supplementary_revised/Supplementary table 5.pdf]

circRNA	log2FC	miscRNA	log2FC	lncRNA	log2FC	piRNA	log2FC
MACF1	1.35	RN7SKP160	0.57	AL117329.1	1.35	piR-30348	0.40
MEF2A	1.20	RN7SKP175	0.43	SNAP25-AS1	1.21	piR-59892	0.34
THBS1	1.17	RN7SKP193	0.40	AL590438.1	1.13	piR-36372	0.32
TBCCD1	1.17	RN7SKP187	0.38	AL133313.1	1.01	piR-58356	0.04
METTL3	1.17	RN7SL787P	0.33	USP2-AS1	0.99	piR-38600	-0.01
SLC39A10	1.16	RN7SL3	-1.16	UBL7-AS1	-1.47	piR-60126	-0.03
SEC31B	-1.38	RN7SKP80	-1.21	PACRG-AS3	-1.47	piR-33382	-1.18
GOLGA3	-1.38	RN7SL2	-1.38	AC092338.3	-1.50	piR-36376	-1.20
GRIN2D	-1.40	RN7SL4P	-1.44	C8orf34-AS1	-1.51	piR-56450	-1.22
WDR67	-1.42	RN7SL5P	-1.80	LINC01297	-1.52	piR-31985	-1.25
PSEN2	-1.48			AD000090.1	-1.54	piR-36063	-1.36
				AL732437.2	-1.83	piR-52016	-1.54

Table S5: Differentially regulated non-coding RNAs identified using miRMaster2.0.
