# Supplementary material for: Paired Transcriptomic Analyses of Atheromatous and Control Vessels Reveal Novel Autophagy and Immunoregulatory Genes in Peripheral Artery Disease
Source: Cells. 2024 Jul 28;13(15):1269. doi: 10.3390/cells13151269 (PMC11312159; doi:10.3390/cells13151269)
Supplement: Supplementary file 1 [file cells-13-01269-s001.zip › Supplementary_revised/Supplementary table 6.pdf]

Cell Type	Downregulated Enriched cell subtypes	Upregulated Enriched cell subtypes
Endothelium	3	2
Epithelium	7	7
Immune cells	26	20
Stromal cells	2	1
Stem cells and progenitors	9	10
Germ cells	7	8
Other	5	8
Total	59	56

Table S6: Cell imputation in plaque tissue: Number of cell types in plaque samples was imputed using Cellmarker database using differentially expressed genes. The cell types were then manually curated into broad cell subtypes as mentioned above.
