# Supplementary material for: Paired Transcriptomic Analyses of Atheromatous and Control Vessels Reveal Novel Autophagy and Immunoregulatory Genes in Peripheral Artery Disease
Source: Cells. 2024 Jul 28;13(15):1269. doi: 10.3390/cells13151269 (PMC11312159; doi:10.3390/cells13151269)
Supplement: Supplementary file 1 [file cells-13-01269-s001.zip › Supplementary_revised/Supplementary table 7.pdf]

	Identified from downregulated genes			Identified from upregulated genes		
**Cell types**	**Mapped genes**	**Total Markers**	**Gene enrichment**	**Mapped genes**	**Total marker genes**	**Gene enrichment**
Endothelial	16	939	1.7E-02	10	844	1.2E-02
Epithelial	23	1235	1.9E-02	25	1514	1.7E-02
Immune	234	11170	2.1E-02	183	10675	1.7E-02
Stromal	2	61	3.3E-02	2	61	3.3E-02
Stem cell + Progenitors	29	1359	2.1E-02	23	1016	2.3E-02
Germ cells	79	4153	1.9E-02	71	4235	1.7E-02
Others	18	1268	1.4E-02	39	1639	2.4E-02

Table S7: Cell type composition of plaque tissue: Differentially expressed genes were used to predict cell types involved in plaque tissues. Identifications were classified into 7 major cell classes and mapped genes were identified. Gene enrichment is the ratio of mapped genes to total markers in the database.
