# Supplementary material for: Paired Transcriptomic Analyses of Atheromatous and Control Vessels Reveal Novel Autophagy and Immunoregulatory Genes in Peripheral Artery Disease
Source: Cells. 2024 Jul 28;13(15):1269. doi: 10.3390/cells13151269 (PMC11312159; doi:10.3390/cells13151269)
Supplement: Supplementary file 1 [file cells-13-01269-s001.zip › Supplementary_revised/Supplementary table 8.pdf]

**Gene name**	**Forward primer sequence (5'-3')**	**Reverse primer sequence (5'-3')**
AP3B1	GTGAAGGGAAAGGCACTGGA	GCCACAAACTCTTCTGGGGT
LYRM1	CCGGAAAAACAAAAATCTCACGG	GAGTGGGGTAAGGCCCATTG
USP8	CCTTCTAAACCTGCTGCCCA	GCAGCAACTGGTTCAACTGG
NLRP1	CAACAAGACTTGAACACAACGAG	CTCTCAATGACTGTGCTGGGTA
Rab11	ACGTCATCTCAGGGCAGTTC	AGAAACAATGCGGTAAATCTCTGT
SAR1B	GTTCAAGCTCGAAGAGTGTGG	GGTCTGCACAATCCACCAGA
ULK2	TTTAAATACAGAACGACCAATGGA	GGAGGTGCCAGAACACCA
E2F3	CCAAAAACTCCAAAATCTCCCTCA	GCACTTCTGCTGCCTTGTTC
ATG3	GTGAAGGGAAAGGCACTGGA	GCCACAAACTCTTCTGGGGT
FGFR3	TGCTGAATGCCTCCCACG	CGTCTTCGTCATCTCCCGAG
LOXL1	CATGGACGAGTTCAGCCACT	TTGAGGTTGCCGAAGTCACA

Table S8: Genes and corresponding primer sequences used for validation of transcriptomic data.
