# Supplementary figures and images for: Paired Transcriptomic Analyses of Atheromatous and Control Vessels Reveal Novel Autophagy and Immunoregulatory Genes in Peripheral Artery Disease
Source: Cells. 2024 Jul 28;13(15):1269. doi: 10.3390/cells13151269 (PMC11312159; doi:10.3390/cells13151269)

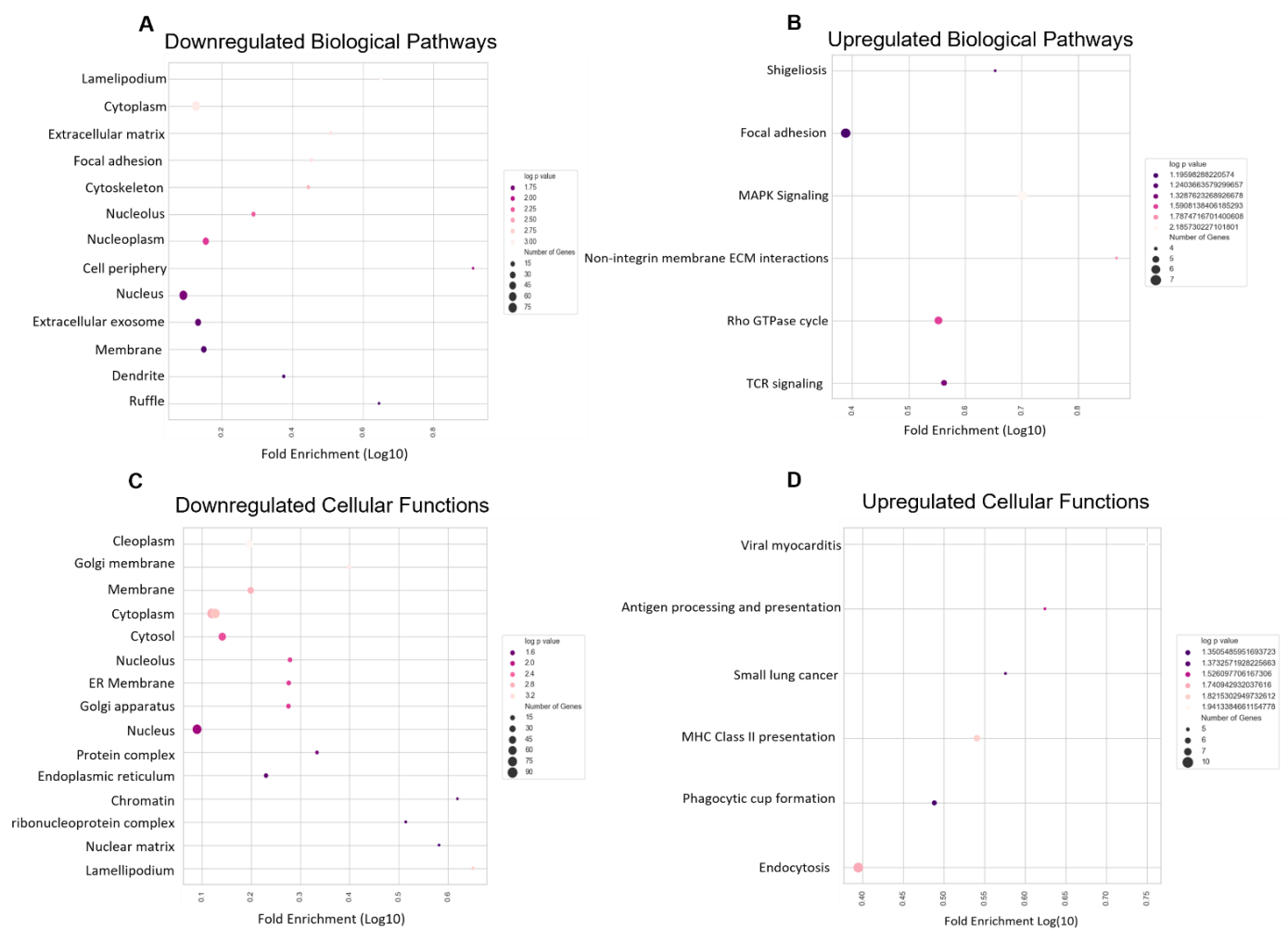

Figure S2: Top dysregulated biological pathways and cellular components in PAD.

Supplement: Supplementary file 1 [file cells-13-01269-s001.zip › Supplementary_revised/Supplementary figure 2.pdf]
